# The potential regulatory role of the lncRNA-miRNA-mRNA axis in teleost fish

**DOI:** 10.3389/fimmu.2023.1065357

**Published:** 2023-02-21

**Authors:** Zhixia Zhou, Cuibo Leng, Zhan Wang, Linhai Long, Yiju Lv, Ziru Gao, Yin Wang, Shoushi Wang, Peifeng Li

**Affiliations:** ^1^ Institute for Translational Medicine, The Affiliated Hospital of Qingdao University, College of Medicine, Qingdao University, Qingdao, China; ^2^ The Affiliated Qingdao Central Hospital of Qingdao University, The Second Affiliated Hospital of Medical College of Qingdao University, Qingdao, China

**Keywords:** lncRNA, miRNA, teleost, reproduction, immunity, infection

## Abstract

Research over the past two decades has confirmed that noncoding RNAs (ncRNAs), which are abundant in cells from yeast to vertebrates, are no longer “junk” transcripts but functional regulators that can mediate various cellular and physiological processes. The dysregulation of ncRNAs is closely related to the imbalance of cellular homeostasis and the occurrence and development of various diseases. In mammals, ncRNAs, such as long noncoding RNAs (lncRNAs) and microRNAs (miRNAs), have been shown to serve as biomarkers and intervention targets in growth, development, immunity, and disease progression. The regulatory functions of lncRNAs on gene expression are usually mediated by crosstalk with miRNAs. The most predominant mode of lncRNA-miRNA crosstalk is the lncRNA-miRNA-mRNA axis, in which lncRNAs act as competing endogenous RNAs (ceRNAs). Compared to mammals, little attention has been given to the role and mechanism of the lncRNA-miRNA-mRNA axis in teleost species. In this review, we provide current knowledge about the teleost lncRNA-miRNA-mRNA axis, focusing on its physiological and pathological regulation in growth and development, reproduction, skeletal muscle, immunity to bacterial and viral infections, and other stress-related immune responses. Herein, we also explored the potential application of the lncRNA-miRNA-mRNA axis in the aquaculture industry. These findings contribute to an enhanced understanding of ncRNA and ncRNA-ncRNA crosstalk in fish biology to improve aquaculture productivity, fish health and quality.

## 1 Introduction

Over the past two decades, the primary function of RNA is no longer what was once thought to be a mere intermediate molecule of genetic information from DNA to protein because the RNA pool contains thousands of noncoding RNA (ncRNA) transcripts that have little or no ability to form proteins (1, 2). Although ncRNAs are not directly involved in gene coding and protein synthesis, they can act as regulators to regulate gene expression at the epigenetic, transcriptional, posttranscriptional, translational, and posttranslational levels (3, 4). Therefore, an increasing number of ncRNAs, especially microRNAs (miRNAs) and long noncoding RNAs (lncRNAs), have been identified for their important roles in cellular physiology or pathological processes in various species, including teleost fish (5–7).

miRNAs, one of the most abundant and most studied natural single-stranded small ncRNAs, are 21 to 24 nucleotides in length and are generally highly conserved from yeast to vertebrates (8). miRNA can bind to the 3’- and 5′-untranslated region (UTR), promoter region and coding region of the messenger RNA (mRNA) target by base-pairing with complementary sites, thereby inhibiting the translation of mRNA into protein or inducing mRNA degradation (9, 10). In mammals, more than 60% of mRNAs have been predicted to be regulated by miRNAs, which are involved not only in various physiological processes but also in the pathophysiological processes of various diseases (11–13). In teleost fish, miRNAs were first discovered in zebrafish (*Danio rerio*, *D. rerio*) and were soon identified in various fish species, such as rainbow trout (*Oncorhynchus mykiss*, *O*. *mykiss*), bighead carp (*Aristichthys nobilis*, *A. nobilis*), silver carp (*Hypophthalmichthys molitrix*, *H. molitrix*), common carp (*Cyprinus carpio*, *C. carpio*), channel catfish (*Ictalurus punctatus*, *I. punctatus*), flounder (*Paralichthys olivaceus*, *P. olivaceus*) and large yellow croaker (*Larimichtly crocea*, *L. crocea*) (14–17). Similar to mammals, fish miRNAs have also been shown to be involved in fish development, nutrition, immune and inflammatory responses, and their roles and molecular mechanisms have gradually been revealed (15, 18–22).

**Figure 1:**
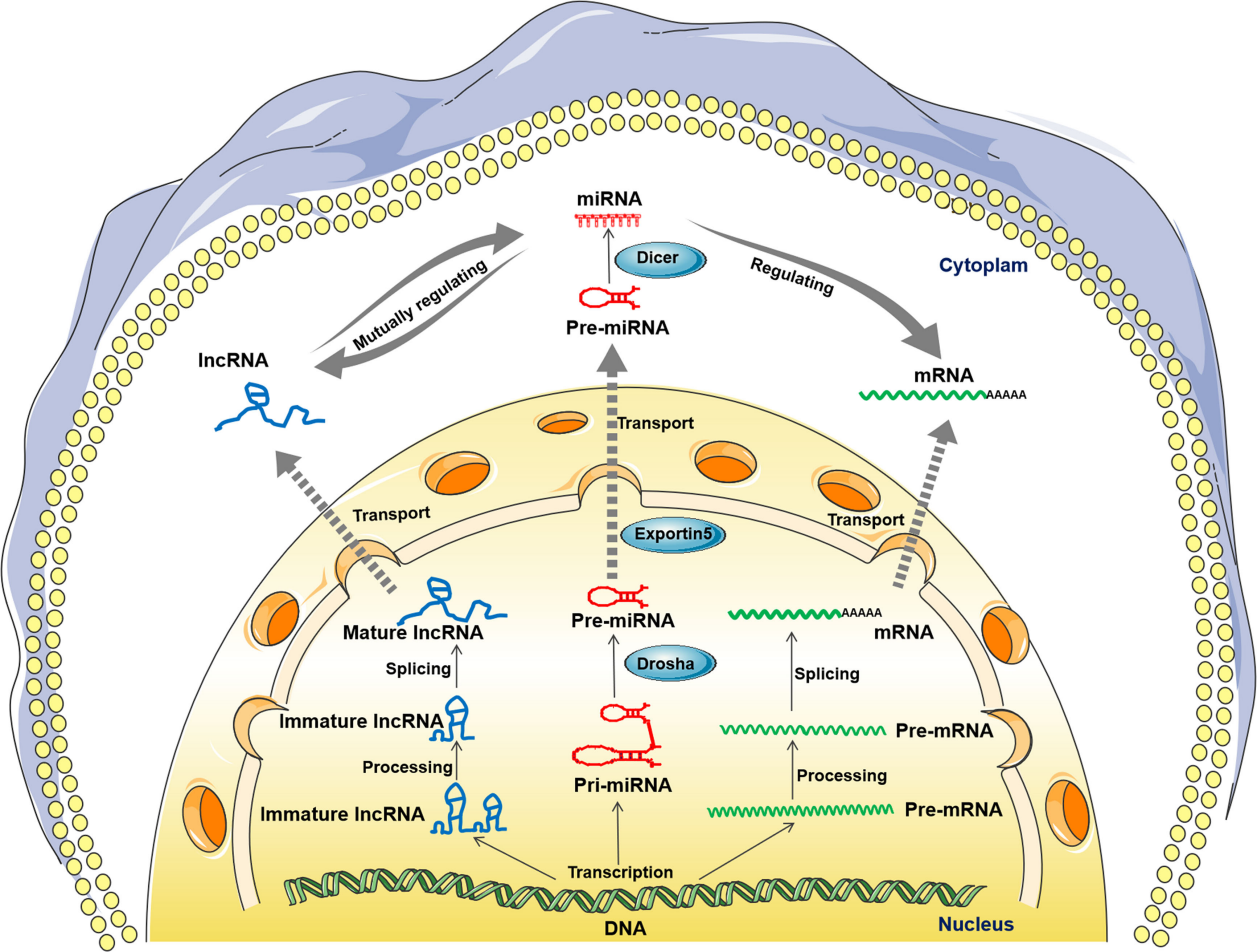
Biogenesis of the long noncoding RNA (lncRNA)-microRNA (miRNA)-messenger RNA (mRNA) axis. (lncRNA, left) Immature lncRNAs are transcribed from the intergene, exon or distal protein coding region of the genome and become stable immature lncRNAs after posttranscriptional processing. Then, most lncRNAs undergo alternative splicing and become mature lncRNAs. Mature lncRNAs can exist in the nucleus or be transported to the cytoplasm. (miRNA, middle) Primary miRNAs (pri-miRNAs) transcribed from the genome are converted into precursor miRNAs (pre-miRNAs) by Drosha cleavage. Then, the premiRNAs were transferred to the cytoplasm in an Exportin-5-dependent manner. Finally, mature miRNAs are produced from premiRNAs after Dicer1 cleavage. (mRNA, right) The initial transcription products of mRNAs are high molecular weight precursor RNAs (pre-mRNAs). Then, the premRNAs are transformed into mature mRNA after posttranscriptional processing, alternative splicing and RNA modification. Subsequently, the mRNAs are transported into the cytoplasm and translated. In the cytoplasm, lncRNA can interact with miRNA, acting mainly on miRNA as a competing endogenous RNA (ceRNA), thus regulating its target molecule mRNA and inducing its translation repression or decoy.

lncRNA is another of the most widely studied ncRNAs with lengths generally more than 200 nt, which plays an important role in growth and development, and its dysregulation is associated with a variety of diseases (23, 24). Compared with miRNAs, most lncRNAs have lower sequence conservation across species, but they can regulate gene expression at almost all levels (25). In addition, probably because of their lower conservation, their expression patterns in cells or tissues are more specific than miRNAs or even mRNAs (19). lncRNAs act as molecular signaling activators, decoys, guides, or scaffolds that interact with a range of DNAs, RNAs, and proteins to influence their function, especially miRNAs (23, 26–29). At least four patterns of lncRNA-miRNA interactions have been identified in mammals as follows: 1) lncRNAs act as competing endogenous RNAs (ceRNA)/sponges or decoys to bind miRNAs and release their target mRNAs; 2) lncRNAs as precursors are one of the sources of miRNAs; 3) lncRNAs compete with miRNAs to bind directly to mRNAs; and 4) miRNAs induce the degradation of lncRNAs (19, 29–31). Among these RNAs, the first is the most predominant mode of interaction between lncRNAs and miRNAs, which is termed the lncRNA-miRNA-mRNA ceRNA network mode, as shown in **Figure 1**. Undoubtedly, most lncRNA functions are mediated by their ceRNA networks. In fish, similar to miRNAs, lncRNAs have also been found in a variety of fish and have been shown to be involved in fish liver metabolism (32, 33), growth and development (34–38), as well as immune responses to various stresses, such as netting and chasing (39), hypoxia (40), bacteria (40–43), and viruses (44, 45). However, compared with fish miRNAs, the identification, function and mechanisms of fish lncRNAs are still relatively poorly studied, including the signaling pathways associated with the lncRNA-miRNA-mRNA regulatory network.

Herein, we provide a brief overview of recent advances in the regulatory roles of the lncRNA-miRNA-mRNA network in teleost fish physiological and pathological processes, mainly including teleost growth and development, reproduction, immune response to infection, and other immune-related biological processes. Furthermore, we explored the potential application of the lncRNA-miRNA-mRNA axis as a biomarker or intervention target in fish domestication, farming or disease treatment. This may help to elucidate the fish lifestyle related to the regulation of the lncRNA-miRNA-mRNA network and provide new ideas for ncRNA, targeting fish growth, breeding, or disease treatment strategies to improve the sustainable development of fisheries.

## 2 The regulatory roles of the lncRNA-miRNA-mRNA axis in teleost physiology and pathology

**Figure 2:**
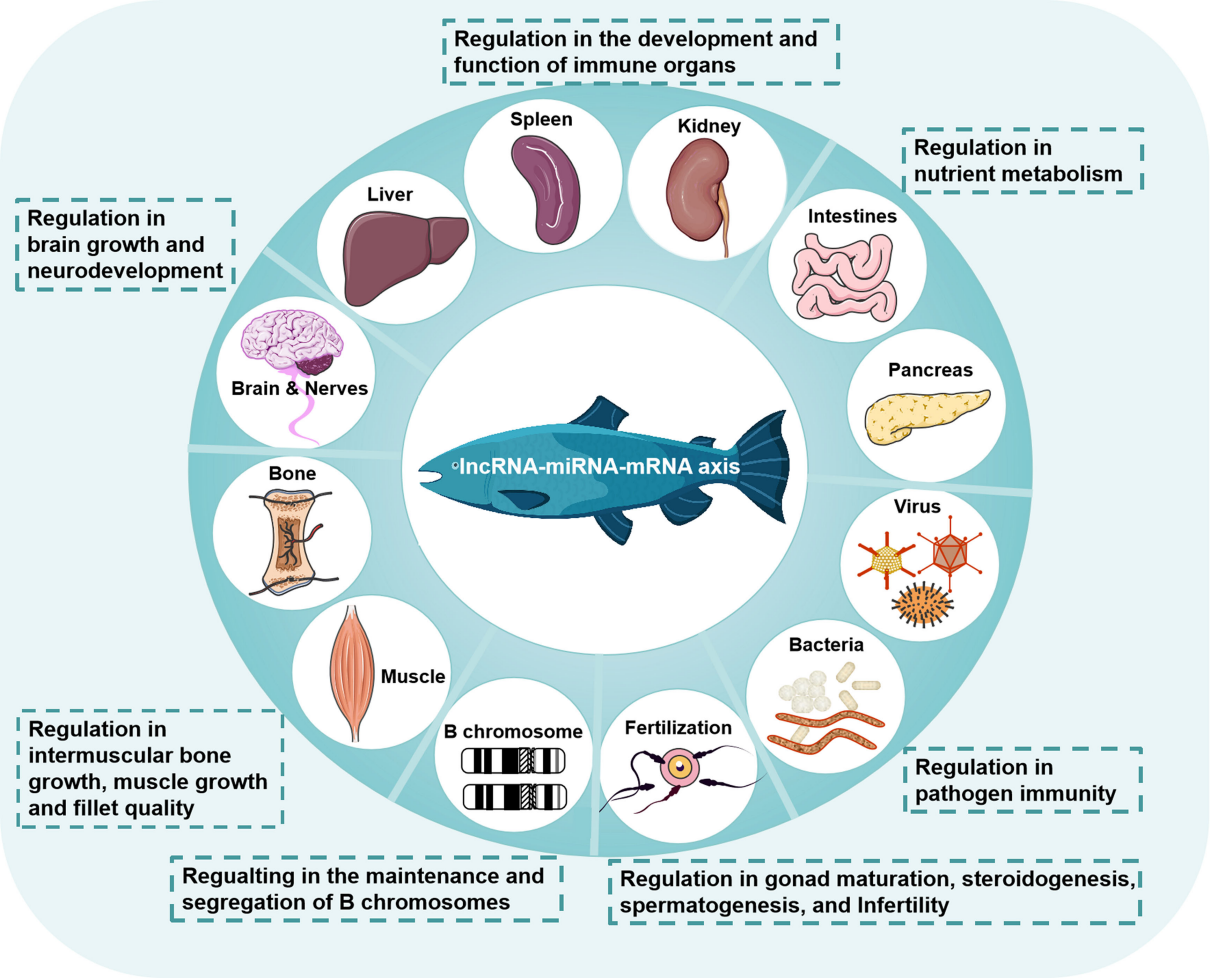
Regulation of the lncRNA-miRNA-mRNA axis in teleost fish. The lncRNA-miRNA-mRNA axis is widely involved in the physiological and pathological processes of fish, including the development and function of immune organs, nutrient metabolism, brain growth and neurodevelopment, intermuscular bone growth, muscle growth and fillet quality, the maintenance and segregation of B chromosomes, gonad maturation, steroidogenesis, spermatogenesis, infertility, and pathogen immunity.

Similar to mammals, the regulatory functions of miRNA-related lncRNAs in fish are mediated mainly by the lncRNA-miRNA-mRNA axis. Different axes have been found in different tissues of different fish species, involving many lncRNAs and miRNAs, which may further suggest the specificity advantage of less conserved lncRNAs. The lncRNAs can be constitutively present in normal cells to maintain cellular homeostasis or regulated in specific stress responses and diseases to induce innate or specific immunity, as shown in **Table 1** and **Figure 2**.

**Table 1:** LncRNA-miRNA-mRNA axis regulation in teleost fish.

Regulated biological process	Related fish species	Related Organs/Cells	Stimulus	Mainly related lncRNAs	Mainly related miRNAs	Mainly related genes	Mainly related signaling pathways	Refs
Growth and development	*Ctenopharyngodon* *idella*	Brain	None	MSTRG.6764; MSTRG.50349	miR-27a-3p; miR-206	*lpxn*; *axpm*	Actin cytoskeleton; Ras; Chemokine; Immunity	(46)
	*Danio rerio*	Embryos	None	Cyrano	miR-7	UN	Neurodevelopment	(47)
	*Ctenopharyngodon* *idella*	Hepatopancreas	None	MSTRG.35807; MSTRG.21503; MSTRG.25056; MSTRG.41999	miR-13b-5p; miR-22a-5; miR-10b-5p	*cel*; *amy2a*; *repe2*; *cbp1*; *celf1*; *ela3l*; *cpa2*	Biosynthesis; Immunity; Pancreatic secretion; Peroxisome; Ras; Nutrient metabolism	(46)
	*Oncorhynchus* *kisutch*	Liver; Kidney; Spleen	None	NL	NL	NL	TGF-β; NF-κB; Cytokines; Immunity; Adherens junction	(48)
	*Paralichthys* *olivaceus*	Gill; Liver; Kidney; Intestine	None	NL	NL	NL	Antigen processing and presentation; TLRs; Immunity	(49)
	*Paralichthys* *olivaceus*	Skeletal muscle	None	TCONS_00003213; TCONS_00006684; TCONS_00023918	miR-133-5p; miR-221-3p; miR-124-5p;	GS-010675; GS-018639; GS-016120	Actin cytoskeleton; Tight junction; Focal adhesion	(50)
	*Sparus aurata*	Fast skeletal muscle	None	lncRNA20194	miR-133; miR-206; miR-20	*myod1*	Myoblast proliferation	(51)
	*Oncorhynchus mykiss*	Muscle	None	Omy500041161; Omy400178299	mir-26a; mir-4185; mir-10b-mature 3'; mir-181d-mature 5'	GSONMT00080511001; GSONMT00041090001	TGF-β; Protein catabolism/anabolism; Immunity	(52)
	*Megalobrama amblycephala*	Intermuscular bone	None	LNC_017705; LNC_007210; LNC_011298	miR-24b-3p; miR-193b-3p	*zip1*; *C6*; MamblycephalaGen-e23275	Osteoblast differentiation; Ca2^+^ deposition	(53)
	*Astatotilapia latifasciata*	B chromosomes	None	BncRNA	miR-129-3p; miR-9-5p; miR-153a-5p	UN	Maintenance and segregation of B chromosome	(54)
Reproduction	*Diplodus puntazzo*	Infemale gonads	None	DP-novel-07606; DP-novel-06984; DP-novel-07767	miR-122-1; miR-7a; miR-129; miR-125c	XR_003984576.1; XR_003429050.1; XR_001814721.1	Gonad maturation	(55)
	*Paralichthys olivaceus*	Ovary; Testis	None	TCONS-00021450; TCONS-00058013; TCONS-00058894	miR-20a; let-7; miR-82155_166	*dnah1*; *dnah11*; *dnah12*	Steroidogenesis; Spermatogenesis	(56)
	*Paralichthys olivaceus*	Ovary; Liver	None	LNC-001695; LNC-007947; LNC-002362; LNC-003418	novel_103; novel_120; novel_125; novel_167	ARMC6; PCCA; TANC2; TNR5; MMP28	Steroid biosynthesis; Metabolism; Immunity; Signal transduction	(57)
Bacterial infection	*Salmo salar;* *Sebastes schlegelii*	Gill; Spleen	*Aeromonas salmonicida*	TCONS_00079020; LOC106564649; TCONS_00022856; LNC_00116154	miR-155-5p; miR-551-3p; miR-8157-3p; novel_264	*atm*; *tp53*; *mdm4*; NLRC3	P53; Wnt/β-catenin; mTOR; Metabolism; Immunity; Phagocytosis; TLRs; Degradation	(58, 59)
	*Paralichthys olivaceus;* *Miichthys miiuy;* *Nibea diacanthus;* *Larimichthys crocea*	Spleen; MICs; EPCs; Kidney cells	*Vibrio anguillarum*	lncRNA-IRL	miR-27c-3p	IRAK4	Pathogen recognition and killing; NF-κB; Apoptosis; Immunity	(60, 61)
	*Paralichthys olivaceus*	Intestine; HEK293T	*Edwardsiella tarda*	LNC_001979	novel_171	*potusc2*; *podad1*	Autophagy; PPAR, Endocytosis; MAPK, Notch; Phagosomes; Immunity	(62)
	*Ctenopharyngodon idella*	Spleen; Muscle; Brain; Heart; Fin; Gill; CIKs	*Aeromonas hydrophila*	lncRNA-WAS; lncRNA-C8807; lncRNA-SUMO3; lncRNA-HDMO13; LncRNA-ANAPC2; lncRNA-NEFM	miR-142a-3p; miR-21; miR-451	*ccr7*; *glut3*; *jnk*; *tnfaip2*; *npr2*; *hdac8*	NF-kB; MAPK; Immunity	(63–65)
	*Oreochromis niloticus*	Spleen; Kidney	*Streptococcus agalactiae*	MSTRG.2496.2; MSTRG.204071.1; MSTRG.61707.9; MSTRG.129013.3	miR-265; miR-574; miR-466; miR-2305; miR-7082; miR-4739	ON_73287; ON_86194; PB_14857; PB_1748; PB_10169; EN_09656	Cytokines; TLRs; Endocytosis; Lysosomal metabolic; Immunity	(66)
	*Epinephelus coioides*	Spleen	*Pseudomonas plecoglossicida*	c115058_g1-i1; c125321_g1-i1; c132960_g1-i1; C202650_g1-i1	miR-731; miR-16b; miR-375; miR-15a-3p	*cela1*; *cela2*; *ctrb*; *prss*	Immunity; Chemokine; Cytokine; Antigen processing and presentation	(67–70)
Viral infection	*Miichthys miiuy*	Liver; Spleen, Kidney; Brain, Heart; Gill; Intestine; MICs	SCRV	AANCR; MARL; NOD1; NARL; MIR2187HG; MIR122HG	miR-210; miR-122; miR-217-5p; miR-2187-3p; miR-122-5p	MITA; MAVS; NOD1; TBK1; TAK1	Cell proliferation; NF-κB; IRF3; Immunity	(71–75)
	*Salmo salar*	Gill	POMV	MSTRG.13941.1	miRNA-30e-3-3p	*selja*	Selenium transport	(76)
Others	*Oreochromis niloticus;* *Oncorhynchus mykiss*	Brain; Skin; Heart; Gill; Intestine; Head kidney; ......	Hypoxia; Cold; Alkalinity; Salinity; Acute heat; ......	TCONS_00151992; MSTRG.11484.2; MSTRG.32014.1; MSTRG.29012.1	miR-128-5p; C-5p-43254_34; PC-3p-28352_70; bta-miR-11987_L-1R-1_1ss8TA	*ifih1*; *dhx58*; *irf3*	MAPK; Phagosome; Immunity; NOD-like receptor; RIG-I-like receptor, ......	(77–79)

NL, not listed; UN, unknown.

### 2.1 Regulation in growth and development

#### 2.1.1 Brain and Nerves

Different expression profiles of mRNA, lncRNA and miRNA were identified in the brain of grass carp (*Ctenopharyngodon Idella*, *C. Idella) at* different growth rates by whole transcriptome sequencing (80). The hub mRNAs and hub ncRNAs in the brain with increased growth rates were associated with many biological processes, including immune, endocrine, and growth hormone signaling pathways, and processes such as amino acid and carbohydrate metabolism. In the lncRNA-miRNA-mRNA ceRNA networks of the brain based on hub mRNAs and hub ncRNAs, the MSTRG.6764-miR-27a-3p-lpxn (leupaxin) and MSTRG.50349-miR-206-axpm (abnormal spindle-like microcephaly associated) axes were identified. All lncRNAs and mRNAs in these two ceRNA networks were determined to be upregulated, while all miRNAs were downregulated. The expression levels of these lncRNAs and mRNAs were further confirmed to be positively correlated with the growth of *C. Idella*, while miR-27a-3p or miR-206 expression was negatively correlated with *C. Idella* growth (80). These data suggest that the difference in grass carp growth rate may be related to the development of the central nervous system of the brain regulated by the ceRNA network.

In addition, recent studies have found that lncRNAs can not only serve as miRNA precursors or ceRNAs but can also be silenced as miRNA targets in fish (46). Lee reported that miRNAs primarily targeted and silenced the 5’ caps and 3’ poly(A)-tails of lncRNAs in *D. rerio* at the posttranscriptional and translational levels. The mRNA-like lncRNA (containing a 5’ cap, 3’ poly(A)-tail, and canonical miRNA target sites) was inhibited by miRNA at both the RNA and ribosomal levels during the early developmental stages of *D. rerio* embryos, whereas the nonmRNA-like long noncoding RNA (containing miRNA target sites, but no 5’ cap and 3’ poly(A)-tail) was not strongly inhibited by miRNA and may act as a miRNA decoy (46, 59). Cyrano is a miRNA-regulated lncRNA whose expression and function are repressed by miR-7 (59). Cyrano was confirmed to be parentally inherited lncRNA in *D. rerio* and was proven to play an important role in the symmetric development of neuronal structures by interacting with miR-7 (59). In addition, knockdown of Cyrano without altering zygotic Cyrano resulted in changes in zebrafish brain morphology, suggesting that inherited Cyrano is critical for brain development (59). These data indicated that fish lncRNAs and miRNAs have a reciprocal regulatory relationship, which in turn affects the expression and function of growth- or development-related mRNAs.

#### 2.1.2 Immune organs

Similarly, the expression and interaction of lncRNAs, miRNAs and mRNAs was explored in the hepatopancreas, gill, intestine, liver, kidney, and spleen in fish by using high-throughput sequencing technology (47, 80, 81). A total of 10,270 lncRNAs and 7720 mRNAs were identified in gill, intestine, liver and kidney tissues of *P. olivaceus*. The mRNA-miRNA interaction network confirmed that one-third of mRNAs were predicted to be targeted for regulation by more than one miRNA (47). In contrast, 975 lncRNAs and 163 mature miRNAs were found in the liver, kidney, and spleen of coho salmon (*Oncorhynchus kisutch*, *O*. *kisutch*). Among these RNAs, 1339 lncRNAs putatively interacted with 148 miRNAs, while 6 lncRNAs were identified as precursors of 16 miRNAs (81). These data support that ceRNAs are the most common mode of relationship between ncRNAs. The central ceRNA networks of the hepatopancreas are formed by 4 lncRNAs, 3 miRNAs and 7 mRNAs in grass carp (80). Fourteen hub lncRNAs and 28 hub miRNAs were both expressed in the liver and head kidney of coho salmon (81). This finding indicates that the two main immune organs of fish have relatively similar ncRNA expression patterns, supporting ncRNAs as biomarkers of tissue/organ-specific expression and developmental stages of organisms (48, 81). Moreover, the pathways of the target genes of these ncRNAs are concentrated in 12 pathways, such as the transforming growth factor-beta (TGF-β) signaling pathway, cytokine-cytokine receptor interaction, adherens junction, and nuclear transcription factor (NF)-κB signaling pathway (81), suggesting that ncRNAs and their interactions play important regulatory roles in the development or function of fish immune organs.

#### 2.1.3 Skeletal muscles

Regarding skeletal muscle, different expression profiles of lncRNAs and mRNAs were identified in different developmental stages of Japanese flounder (62). These lncRNAs were then predicted to take part in skeletal muscle development *via* cis- or trans-acting mechanisms. In addition, coexpression networks of the lncRNA-miRNA-mRNA axis showed that most lncRNAs interact with one or two predicted miRNAs. Some lncRNAs can even interact with at least three target miRNAs, such as TCONS_00093971, TCONS_00096817, and TCONS_00032744 (62). Similarly, a total of 290 lncRNAs are differentially expressed in juvenile and adult fast skeletal muscle of gilthead sea bream (*Sparus aurata*, *S. aurata*), and the number of differential lncRNAs is greater in juveniles than in adults, indicating that most of the differential lncRNAs play a role in the muscle growth of juveniles (49). In addition, most of the differential lncRNAs (such as lncRNA20194) were more active in myoblast proliferation and were downregulated during the fusion process, which may play a promoting role in myoblast proliferation by acting as sponges for miR-133, miR-206 and miR-208 (49). Moreover, the different expression profiles of mRNAs and lncRNAs were identified in fish families with different phenotypes of rainbow trout (49, 82). In the constructed lncRNA-miRNA-mRNA network, 3 lncRNAs (Omy500041161, Omy400178299, and Omy500089619) were coexpressed with mRNAs known to be associated with whole body weight (WBW), muscle yield and fat content, such as lipoprotein lipase (LIPL) and TGF-β, to impact muscle quality traits. Moreover, 44 lncRNAs were able to interact with miRNAs as sponges to control mRNAs belonging to protein catabolic/anabolic pathways, affecting muscle mass characteristics and fast/effective growth rates (82). These data demonstrate the regulatory role of lncRNAs and their ceRNA networks in muscle growth and development and in the characteristics of muscle growth and fillet quality.

#### 2.1.4 Intermuscular bone

Intermuscular bone (IB) is a small spicul-like bone present in the muscular septum of teleost fish that adversely affects the food and economic value of fish (83). The expression profiles of mRNAs and ncRNAs (lncRNAs and miRNAs) were different in blunt snout bream (*Megalobrama amblycephala*, *M. amblycephala*) at two intermuscular bone (IB) growth stages (1 and 3 years old) (50). The slow-growing IB-3 was found to possibly be due to the reduced osteoblast differentiation and Ca^2+^ deposition caused by ZIP1 downregulation. In addition, 14 ceRNA axes related to the growth of IBs were identified with 10 lncRNAs, 7 miRNAs, and 10 mRNAs (50). Among these RNAs, dre-miR-24b-3p and dre-miR-193b-3p were confirmed to be core miRNAs that could interact with 4 lncRNAs (LNC_007210, LNC_011298, LNC_001774, and LNC_017705) and 3 mRNAs (iron-regulated transporter (IRT)-like protein (ZIP), complement component 6 (C6), and *M. amblycephala* Gene 23275). In particular, the lnc017705-miR-24a-3p-ZIP1 axis is likely to regulate the development of IB (50), which suggests their regulatory roles in the growth of IB in *M. amblycephala*.

#### 2.1.5 B chromosomes

B chromosomes (Bs) are predominantly found in karyotype species of eukaryotic taxa and are considered extra or redundant chromosomes (84). Recently, Bs in fish were found to also be able to generate lncRNAs, termed B chromosome long noncoding RNA (BncRNA) (67). BncRNA is transcribed from a transcriptionally active repetitive DNA (BncDNA) that is highly expressed on all B chromosomes in the cichlid fish (*Astatotilapia latifasciata*, *A. latifasciata)*. In addition, BncRNA was predicted to be involved in the maintenance and segregation of the B chromosome during cell division by interacting with miRNAs on the B chromosome, including miR-129-3p, miR-9-5p, and miR-153a-5p (67), possibly leading to a new understanding of B chromosomes and lncRNA-miRNA regulatory networks in fish development.

### 2.2 Regulation in reproduction

#### 2.2.1 Gonad maturation

ncRNAs, including lncRNAs, miRNAs, rRNAs, and piRNAs, have been found to show differential expression between mature and immature gonads of infemale sharpsnout seabream (*Diplodus puntazzo*, *D. puntazzo*) (51). The study found that 8 of the 10 lncRNAs that were identified in the National Center for Biotechnology Information (NCBI) basic local alignment search tool (BLAST) hit were highly expressed in immature fish, while 8 of the 10 identified miRNAs were highly expressed in mature fish (51). Furthermore, putative lncRNA-miRNA-mRNA hybridizations were constructed, which included 3 lncRNAs (DP-novel-07606, DP-novel-06984, and DP-novel-07767), 5 miRNAs (miR-122-1, miR-129, miR-125c, miR-7a, and ENSGACT00000282061), and 3 mRNAs (XR_003984576.1, XR_003429050.1, and XR_001814721.1) (51). Most miRNAs in these networks have been proven to be associated with a broad range of physiological processes, including gonad maturation. For example, miR-125c can inhibit the maturation of immature females by inhibiting the expression or proteolysis of vitellogenins and yolk proteins (68). In addition, follicle-stimulating hormone receptor (FSHR) is a target of miR-125c, which is a critical gene for the growth of the primary ovarian follicle (69), suggesting that lncRNAs highly expressed in immature gonads may promote gonad maturation by regulating the miRNA-mRNA pathway.

#### 2.2.2 Steroidogenesis and Spermatogenesis

Similarly, ncRNAs also showed differential expression between gonads of different sexes in gynogenetic Japanese flounder. A total of 6772 differentially expressed mRNAs (3541 testis-biased and 3231 ovary-biased), 2284 lncRNAs (1870 testis-biased and 414 ovary-biased), and 244 miRNAs (146 testis-biased and 98 ovary-biased) were obtained between gynoenetic female ovaries and sex-reversed neomale testes (52). Clearly, the numbers of differentially expressed mRNAs and lncRNAs were significantly higher in the testis than in the ovaries, suggesting that ncRNAs function more actively in the neomale testis, especially lncRNAs. Furthermore, the lncRNA-miRNA-mRNA interaction network was constructed with 91 mRNAs, 64 lncRNAs, and 98 miRNAs. In this network, some hub miRNAs interact with many lncRNAs. For example, ovary-biased let-7 binds to 18 lncRNAs and targets dnah1, testis-biased miR-20a interacts with 14 DElncRNAs and targets dnah11, while the novel miRNA-82155_166 can cooperate with 57 lncRNAs to target most large mRNAs. These miRNA-associated regulatory axes involve numerous steroid biogenesis- and sperm motility-related genes and pathways, such as the cytoskeleton, microtubule cytoskeleton, cytoplasmic dynein complex, tubulin and actin binding (52). These data suggest the regulatory roles of the lncRNA-miRNA-mRNA network in steroidogenesis and sexual spermatogenesis in *P. olivaceus*.

#### 2.2.3 Infertility

Moreover, the lncRNA-miRNA-mRNA ceRNA network was proven to be involved in the mechanism underlying infertility in fish. The liver is essential for fish fertility because it can synthesize vitelline and the precursor of vitelline, which is required for oocyte development and maturation (53, 85). Compared with fertile fish livers, sterile fish livers were found to have fewer vacuoles and significantly lower serum vitellogenin levels in *P. olivaceus* (86). Moreover, ncRNAs (mRNAs, lncRNAs, circular RNAs, and miRNAs) were differentially expressed in infertile and fertile individuals *P. olivaceus*). The lncRNA-miRNA-mRNA ceRNA network was constructed with 92 lncRNAs, 14 mRNAs, and 4 miRNAs, which included the identified potentially functional ncRNAs in steroid biosynthesis pathways, such as pol-miR-133-3p, pol-miR-221-3p, XLOC_008437, XLOC_015293, and XLOC_019323. Among the target genes, armadillo repeat-containing protein 6 (ARMC6), propionyl-CoA carboxylase alpha chain (PCCA), tetratricopeptide repeat, ankyrin repeat and coiled-coil containing 2 (TANC2), transposable DNA element 5 (TNR5), and matrix metalloproteinase 28 (MMP28) were associated mainly with metabolism (fat and glycerophospholipid), signal transduction (complement and coagulation cascades), and immunity (RIG-I-like receptor signaling pathway) (86). These results indicate that fish infertility is related not only to histological structure, hormone secretion, and the steroid biosynthesis pathway but also to liver metabolism, immunity, and signal transduction, which may all be regulated by ncRNAs and their ceRNA network.

### 2.3 Regulation in bacterial infection

**Figure 3:**
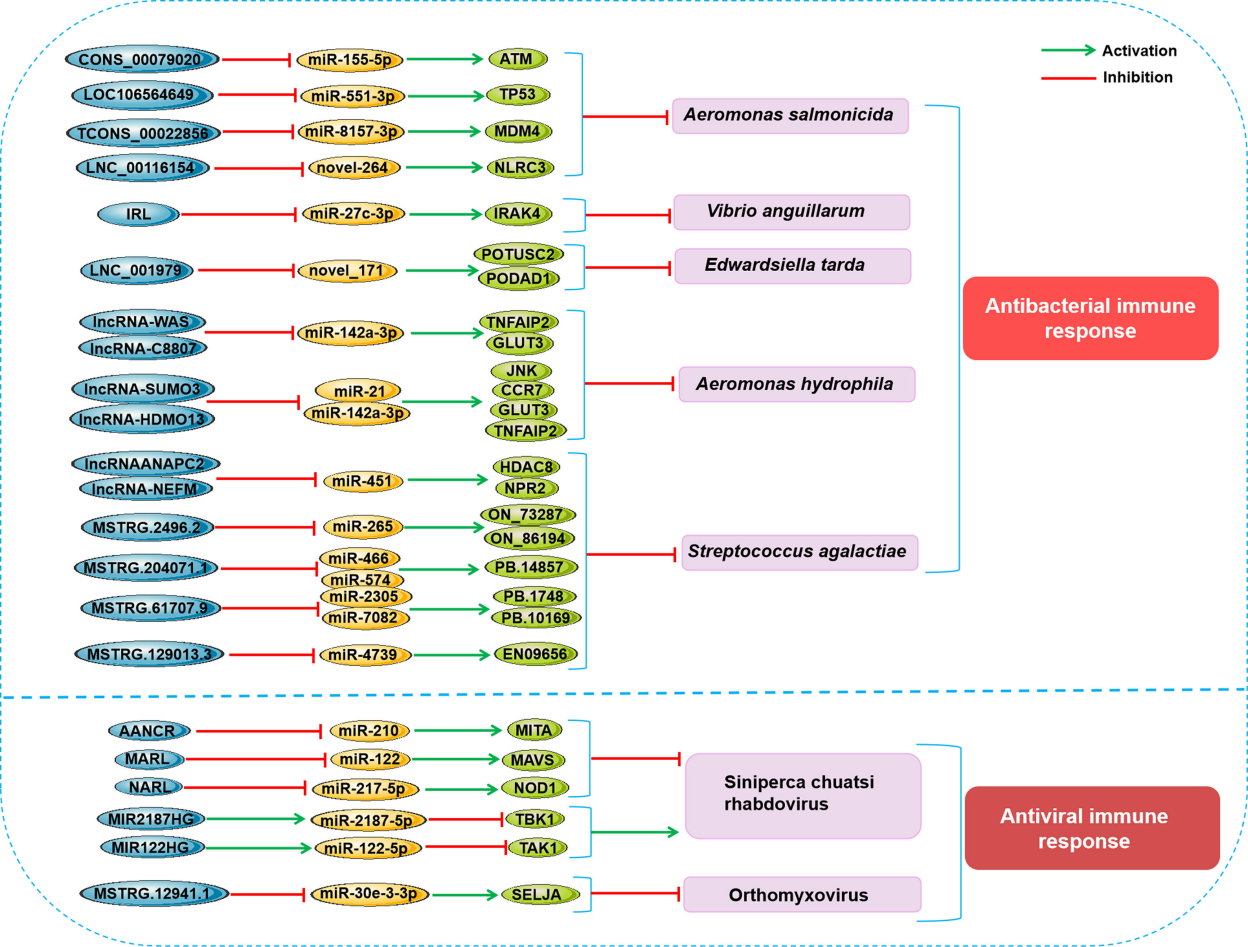
Schematic presentation of the main lncRNA-miRNA-mRNA axes involved in the fish antipathogen immune response. (up) Main lncRNA-miRNA-mRNA axes related to the antibacterial immune response in fish. (down) Main lncRNA-miRNA-mRNA axes related to antiviral immune response in fish.

Infection is a life-and-death struggle between host and pathogen (54), in which host must mobilize its immune system to win (55). Similar to other vertebrates, the immune response of organisms to the invasion of foreign pathogens in teleost fish usually involves the regulation of ncRNAs, especially lncRNAs and miRNAs (19, 20). In fish infected by various types of bacteria or viruses, many immune-related lncRNA-miRNA-mRNA regulatory networks have been identified and play an antibacterial, antiviral or opposite role, as shown in **Figure 3**.

#### 2.3.1 Aeromonas hydrophila (A. hydrophila)

lncRNA-WAS, lncRNA-C8807, lncRNA-SUMO3, lncRNA-HDMO13, lncRNA-ANAPC2 and lncRNA-NEFM were recently identified in grass carp (*C. idella*) and are constitutively expressed in all tested tissues, including the spleen, muscle, brain, heart, fin, and gill (66, 70, 87). However, their expression levels in grass carp kidney (CIK) cells after infection by *A. hydrophila* were found to increase during the initial infection but began to decline and even returned to normal levels later, suggesting their response to *A. hydrophila* infection in cells. Furthermore, overexpression of these lncRNAs was found to significantly promote the cell activity of NF-κB and inflammatory factors, including tumor necrosis factor (TNF)-α, IL-1β, IL-6, IL-8, IL-12, and TGF-β (66, 70, 87). Furthermore, lncRNA-WAS and lncRNA-C8807 were shown to interact with miR-142a-3p to regulate the expression of its targets tnfaip2 and glut3 (70). lncRNA-SUMO3 and lncRNA-HDMO13 may act as sponges to regulate miR-21 and miR-142a-3 to affect their target genes, c-Jun N-terminal kinase (jnk) and CC-chemokine receptor 7 (ccr7), glucosetranspoter isoform (glut3), and tumor necrosis factor alpha induced protein 2 (tnfaip2) (66). LncRNAANAPC2 and lncRNA-NEFM interact with miR-451 to target histone deacetylase 8 (hdac8) and natriuretic peptide receptor 2 (npr2) (87). These network targeting genes tnfaip2, jnk, cc7, glut3, npr2 and hdac8 were all proven to induce inflammatory responses associated with the NF-kB or MAPK pathway (56, 60, 61, 71, 88). These data suggest that the lncRNA-miRNA-mRNA networks mediated by lncRNA-WAS, lncRNA-C8807, lncRNA-SUMO3, lncRNA-HDMO13, lncRNAANAPC2 and lncRNA-NEFM play positive regulatory roles in the inflammatory response of grass carp to *A. hydrophila* challenge.

#### 2.3.2 Aeromonas salmonicida (A. salmonicida)

Based on the identified lncRNAs, miRNAs, and mRNAs that were differentially expressed in gills of Atlantic salmon (*Salmo salar*, *S. salar*) infected with *A. salmonicida*, an immune-related lncRNA-miRNA-mRNA ceRNA network was constructed (72). This network includes 32 lncRNAs, 10 miRNAs, and 16 mRNAs, which are associated with many immune-related signaling pathways, such as p53, (Wnt)/β-chain protein (β-catenin), mammalian target of rapamycin (mTOR), Janus kinase (JAK)/signal transducer and activators of transcription (STAT), and Toll-like receptor (TLR). In addition, three typical lncRNA-miRNA-mRNA axes were found: TCONS_00079020- miR-155-5p- atm (*ataxia telangiectasia*-mutated gene), LOC106564649- miR-551-3p*-* tp53, and TCONS_00022856- miR-8157-3p- mdm4 (murine double minute 4), which were all included in the p53 signaling pathway (85). Meanwhile, 1091 lncRNA-miRNA-mRNA network axes were constructed in black rockfish (*Sebastes schlegelii*, *S.schlegelii*) spleen infected with *A. salmonicida*, which included 400 lncRNAs, 69 miRNAs, and 70 mRNAs (73). The main immune-related signaling pathways involved in the network axis were nutrient metabolism, cell adhesion molecules (CAMs), phagocytosis, and degradation. In the regulatory networks, NLRC3-like genes can be regulated by 17 lncRNAs but only one miRNA (novel_264) at the same time. The key ceRNA triple regulatory network was focused on the LNC_00116154-novel-264-NLRC3 pathway, which may play an important immune regulatory role in the resistance of black rockfish against *A. salmonicida* infection (73). These results indicate the regulatory roles of the lncRNA-miRNA-mRNA network in the immune response of teleost fish against *A. salmonicida* infection.

#### 2.3.3 Edwardsiella tarda (E. tarda)

A total of 115 differentially expressed lncRNAs were identified in the intestine of olive flounder infected by *E. tarda* (89). Among these RNAs, 64 lncRNAs together with 31 miRNAs and 1,766 mRNAs constituted the lncRNA-miRNA-mRNA regulatory network. Many immune-related processes or signaling pathways are involved in the miRNA-mRNA regulatory network mediated by these lncRNAs, such as the signaling pathways of autophagy, peroxisome proliferator-activated receptor (PPAR), endocytosis, mitogen-activated protein kinase (MAPK), Notch, and phagosomes. In addition, two potential ceRNA regulatory networks were preliminarily identified: LNC_001979- novel_171- Potusc2 and LNC_001979- novel_171- Podad1 in the intestine of olive flounder, suggesting their roles in anticeRNA regulatory networks (89). Both tusc2 (tumor suppressor candidate 2) and dad1 (defender against cell death 1) could encode multifunctional proteins associated with cellular processes (57, 90), which suggests that Potusc2- and Podad1-tagged ceRNA regulatory networks may play an important role in regulating the antibacterial immune response against *E. tarda* in olive flounder.

#### 2.3.4 Pseudomonas plecoglossicida (P. plecoglossicida)

In addition, the network of lncRNA-miRNA-mRNA relationships was also proven to be involved in the regulation of immune responses in the spleen of *Epinephelus coioides* (*E. coioides*) infected by *P. plecoglossicida* (63, 64, 91, 92). Compared with the wild-type strain of *P. plecoglossicida*, infection with the L321_RS19110-RNAi (RNA interference) strains, sigX-RNAi strains, L321_20267-RNAi strains, or L321_RS15240-RNAi strains resulted in a delayed onset time, a 20%-50% reduction in the mortality rate of *E. coioides*, and a reduction in symptoms in the spleen, suggesting that L321_RS19110, sigX, and L321_20267, and L321_RS15240 are important disease-causing genes for *P. plecoglossicida* (63, 64, 91, 92). Moreover, these RNAi strains had significant effects on immune-related genes in *P. plecoglossicida-*infected *E. coli*, which were associated with the expression of many lncRNAs. Many upregulated mRNAs are involved in important immune response processes in the spleen, including chemokine or cytokine signaling pathways, receptor-ligand interactions, and antigen processing and presentation (63, 64, 91, 92). For example, chymotrypsin-like elastase family member (cela) 1 was associated with 16 lncRNAs and 4 miRNAs, cela2 was associated with 20 lncRNAs and 5 miRNAs, chymotrypsin B (ctrb) was associated with 24 lncRNAs and 18 miRNAs, and trypsinogen (prss) was associated with 15 lncRNAs and 19 miRNAs (91). In addition, these immune genes are predicted to be regulated by miRNAs and lncRNAs in complex ways, including the formation of lncRNA-miRNA-mRNA ceRNA networks (63, 91, 92). These results suggest that upregulation of immune response-related regulatory lncRNA-miRNA-mRNA networks in *P. plecoglossicida-infected E. coli* may enhance the body’s antibacterial capacity.

#### 2.3.5 Streptococcus agalactiae (S. agalactiae)

In *S. agalactiae*-challenged tilapia (*Oreochromis niloticus*, *O. niloticus)*, 1281 lncRNAs were found to be differentially expressed during infection (65). Among the constructed lncRNA-miRNA-mRNA ceRNA networks, 4 lncRNA (MSTRG.2496.2, MSTRG.204071.1, MSTRG.61707.9, and MSTRG.129013.3)-mediated networks were highly correlated in the spleen and kidney, the main target organs of *S. agalactiae*. In addition, these four lncRNA-mediated ceRNA networks included 6 miRNAs (miR-265, miR-574, miR-466, miR-2305, miR-7082, and miR-4739) and 6 mRNAs (ON_73287, ON_86194, PB_14857, PB_1748, PB_10169, and EN_09656), which were involved in several key immune signaling pathways, such as the cytokine-cytokine receptor interaction pathway, Toll-like receptor signaling, endocytosis pathway, and lysosomal metabolic pathway (65). These data further illustrate the role of lncRNAs and their mediated ceRNA networks in innate immunity against bacterial infection in tilapia.

#### 2.3.6 Vibrio anguillarum (V. anguillarum)

In *Vibrio anguillarum* (*V. anguillarum*)-infected Japanese flounder, 414 lncRNAs were identified that exhibited differential expression, of which 36 lncRNAs acted as competing endogenous RNAs (ceRNAs) interacting with 16 miRNAs and 37 mRNAs (93). Mainly 10 immune pathways were involved in the ceRNA regulatory networks, such as pathogen recognition and killing, NF-κB-regulated inflammation response, apoptosis, and adaptive immunity (93). This finding supports the relationship between the identified ceRNA networks and antibacterial immunity in *V. anguillarum*-treated flounder. Similarly, many lncRNAs were also identified in the spleen tissues of Miiuy croaker (*Miichthys miiuy*, *M. miiuy*) challeged with *V. anguillarum* (94). Interleukin-1 receptor-related kinase (IRAK)-4-related lncRNA (IRL) is one of the significantly upregulated lncRNAs induced by *V. anguillarum*. Knockdown of IRL significantly inhibited the expression levels of tumor necrosis factor (TNF)-α, interleukin (IL)-6, IL-8, and IL-1β in miiuy croaker intestines cells (MICs) upon lipopolysaccharide (LPS) stimulation. In addition, IRL knockdown increased apoptosis but decreased the viability of LPS-treated MICs. Furthermore, IRL was found to act as a sponge for miR-27c-3p to enhance IRAK4 expression, which is a critical regulator involved in the TLR-dependent immune response, thus inhibiting the innate antibacterial response mediated by the production of inflammatory cytokines resulting from activation of the TLR-nuclear transcription factor (NF)-κB signaling pathway. Moreover, the IRL-miR-27c-3p-IRAK4 ceRNA network 4 was shown widely in other teleost fish, including *Nibea diacanthus* (*N. diacanthus*) and *Larimichthys crocea* (*L. crocea*), suggesting the relative conservation of the existence and function of this axis in fish species (94). These results indicate that IRL is a positive regulator of antibacterial responses in fish species by sponging miR-27c-3p.

### 2.4 Regulation of viral infection

#### 2.4.1 Siniperca chuatsi rhabdovirus (SCRV)

The lncRNA-miRNA-mRNA regulation mechanism also exists in the innate antiviral responses of Miiuy croaker to SCRV. Xu reported that a total of 897 lncRNAs were differentially expressed in SCRV-infected spleen samples of miiuy croaker, including the newly identified upregulated lncRNAs antiviral-associated long noncoding RNA (AANCR), mitochondrial antiviral signaling protein (MAVS) antiviral-related lncRNA (MARL), nucleotide oligomerization domain 1 (NOD1) antibacterial and antiviral-related lncRNA (NARL), MIR2187HG and MIR122HG (58, 74, 95–97). Among these RNAs, AANCR, MARL and NARL were subsequently shown to function as positive regulators to counteract the enhancement of SCRV replication. Knockdown of AANCR, MARL, or NARL can promote SCRV replication in SCRV-treated MICs and reduce the expression levels of antiviral-related immune genes, including type I interferon (IFN-1), TNF-α, myxovirus resistance protein 1 (MX1), interferon-stimulated gene 15 (ISG15) and viperin. In addition, their silencing also inhibited cell proliferation in SCRV-treated MICs, whereas their overexpression significantly promoted cell proliferation (74, 95, 96). Mechanistically, AANCR, MARL and NARL were shown to act as ceRNAs by sponging miR-210, miR-122 or miR-217-5p, respectively, to relieve their repressive effects on antiviral gene expression of stimulator of interferon genes (MITA), MAVS or nucleotide oligomerization domain receptor 1 (NOD1), thereby maintaining the stability of the antiviral response of the body and ensuring an appropriate inflammatory response (74, 95, 96). In contrast, MIR2187HG and MIR122HG were proven to be negative regulators to counteract the enhancement of SCRV replication (58, 97). MIR2187HG was identified as a developmental reservoir or as premiR-2187 of miR-2187-3p to increase its expression in SCRV-treated miiuy croaker, thereby inhibiting intracellular TANK-binding kinase 1 (TBK1) expression and TBK1-mediated signaling of NF-κB and interferon regulatory Factor 3 (IRF3) (97). MIR122HG was found to decrease the transforming growth factor-β-activated kinase 1 (TAK1)-triggered NF-κB and IRF3 signaling pathways by acting as a precursor of miR-122-5p (58). These results suggest that the upregulated lncRNAs in miiuy croaker infected by SCRV can positively or negatively regulate the body’s antiviral immune response by interacting with miRNAs, which can not only maintain the stability of the response but also avoid an excessive response to maintain a stable and appropriate antiviral immune response.

#### 2.4.2 Orthomyxovirus (POMV)

In POMV-infected Atlantic salmon, Samsing found that a total of 86 lncRNAs and 478 miRNAs were aberrantly expressed in the gill tissues of infected fish (75). Through the analysis of the ceRNA network constructed between miRNAs, lncRNAs and mRNAs, noncoding RNAs targeting mRNAs were found to be concentrated mainly on genes involved in the immune response process. Among them, the pathway consisting of lncRNA MSTRG.13941.1, miRNA-30e-3-3p and selenoprotein Ja (selja) attracted attention. MSTRG.13941.1 is one of the lncRNAs that rarely changes in fish in the late stage of virus infection. The predicted target genes of miRNA-30e-3-3p include not only fish genes but also virus genes, while the expression level of selja involved in selenium (Se) transport was significantly reduced in late-stage infected fish (75). Selenium is an essential micronutrient for a variety of organisms and has important physiological functions, such as promoting immunity and antioxidation (76), suggesting that the MSTRG.13941.1/miRNA-30e-3-3p/Selja pathway may be involved in virus clearance and homeostasis restoration by regulating selenium metabolism in fish, which requires further study.

### 2.5 Regulation in other stress responses

In an expression analysis of ncRNAs in Nile tilapia (*Oreochromis niloticus*, *O. niloticus*) under at least 15 different tissues and different stress conditions (e.g., hypoxia, cold, alkalinity, salinity, and *Streptococcus agalactiae (S. agalactiae)* infection), 1955 tissue-specific lncRNAs and 99 stress-related lncRNAs were identified (98). Ninety-nine stress-related lncRNAs were predicted to bind to 448 miRNAs, of which 10 lncRNAs contained a motif complementary to 17 mature miRNAs, including the TCONS_00151992-dre-miR-128-5p pair (98). Similarly, when rainbow trout were exposed to acute heat stress, a total of 2605 lncRNAs, 214 miRNAs and 5608 mRNAs were found to be differentially expressed in the head kidney (77). Then, a lncRNA-miRNA-mRNA ceRNA interaction network was constructed based on these differentially expressed lncRNAs, which were significantly enriched in the innate immune response. Immune-related ceRNAs may regulate the acute heat stress-induced response mainly by the MAPK signaling pathway (77). In addition, comparing the whole transcriptome of skin in wild-type rainbow trout, a total of 1630 lncRNAs, 50 miRNAs and 2448 mRNAs were differentially expressed in the skin of yellow mutant rainbow trout, which involved numerous key innate immune-related signaling pathways (78). In the immune-related lncRNA-mediated ceRNA network, the lncRNAs MSTRG.11484.2, MSTRG.32014.1 and MSTRG.29012.1 together with PC-5p-43254_34, PC-3p-28352_70 and bta-miR-11987_L-1R-1_1 ss8TA were identified to regulate at least 3 immune-related genes, interferon-induced helicase C domain-containing protein 1 (ifih1), DEXH (Asp-Glu-X-His) box polypeptide 58 (dhx58) and irf3 (78). These results suggest that the lncRNA-mediated ceRNA network is involved in the fish immune response to various stresses.

## 3 Potential application of the lncRNA-miRNA-mRNA axis in fish

Regarding the above description of lncRNA-miRNA-mRNA interactions in fish, we can speculate that this regulatory mode is highly conserved in biological genetic evolution, although the conservation of lncRNAs is considered to be much lower than the conservation of miRNAs, suggesting that similar to mammals, the lncRNA-miRNA-mRNA axis may also be a potential disease or nondisease biomarker in fish and also suggesting that strategies used in mammals to modulate lncRNAs or miRNAs as molecular targets (silencing or activation) may also be useful in the diagnosis and treatment of fish domestication or disease treatment. Even though lncRNAs are less conserved in fish, targeting the regulation of miRNAs targeted by lncRNA sponges is also a valuable tool for the development of novel therapeutics and high-value biotechnological products for fish. Currently, well-established biotechnologies for lncRNA or miRNA inhibition in mammals include the use of oligonucleotides, RNA interference or small molecule inhibitors, nucleic acid restriction, and clustered regularly interspersed short palindromic repeats (CRISPR)−Cas9 (79, 99–101). In contrast, biotechnologies targeting lncRNA or miRNA overexpression use mainly mimics or viral vector-based gene restoration, transcriptional upregulation, and therapeutic manipulation of ncRNA promoters (102, 103).

In addition, the expression profiles of lncRNAs and mRNAs were found to be altered in the intestine of rainbow trout fed a probiotic diet, suggesting that diet can alter the expression patterns of noncoding RNAs and genes *in vivo* (104). Furthermore, feeding shrimp with bacteria expressing shrimp miR-34 could significantly increase the expression level of miR-34 or mja-miR-35 in shrimp, thereby exerting an antiviral effect against white spot syndrome virus (WSSV) infection. These shrimp were then cooked and fed to a human tumor xenograft mouse model, and the lung metastatic ability of the tumor was significantly reduced in these mice (105, 106). Moreover, dietary miRNA absorption was confirmed to indeed occur in the stomach mediated by mammalian systemic RNA interference defective-1 (SID1) transmembrane family member 1 (SIDT1), which is a key transporter enriched in the stomach (105, 107). These results suggest that dietary or oral administration is an efficient way of delivering ncRNAs *in vivo* for the treatment of diseases such as viral infections or tumors. Using a simple and convenient dietary ncRNA approach to alter nutritional or reproductive strategies regulated by the lncRNA-miRNA-mRNA network would be an effective strategy to improve feeding/breeding efficiency and fishery production.

## 4 Conclusions and discussion

Over the past 20 years, ncRNA-related research has expanded dramatically, especially in the context of human disease. In fish, novel rearing/reproduction strategies targeted by noncoding RNAs have attracted attention to achieve the intriguing goal of optimizing fish growth while maintaining high nutritional value. In recent years, a variety of ncRNAs have been identified and molecules in fish, their functions and molecular mechanisms have gradually become clear, and several fish ncRNA databases have been established, such as FishDB (http://fishdb.ihb.ac.cn) (108). However, compared with the available information for mammals, the amount of information about teleost ncRNAs is still limited. The expression patterns, regulatory functions and molecular mechanisms of ncRNAs in fish need to be further studied. Here, we review the composition and role of the lncRNA-mediated miRNA-mRNA regulatory network in fish growth and development (brain, hepatopancreas, immune organs, skeletal muscles, intermuscular bone, B chromosomes), reproduction (gonad maturation, steroidogenesis, spermatogenesis and infertility), bacterial infection (*A. salmonicida, V. anguillarum, E. tarda, A. hydrophila, S. agalactiae, and E. coioides*), viral infection (SCRV and POMV), and other biological processes, as shown in **Table 1**. These data allow us to better understand the molecular regulatory mechanisms of lncRNA-miRNA-mRNA regulatory networks in fish responses to various environmental stress stimuli and provide new ideas for fish domestication, breeding and feeding, as well as the diagnosis, prevention and treatment of diseases. Interestingly, we found that the regulation of LNC RNA-miRNA-mRNA regulatory networks on the abovementioned fish physiological or pathological processes is basically related to immunity, which further indicates the importance of the fish immune system and immune response in fish growth and development.

However, many lncRNA-miRNA-mRNA axes are limited to the prediction of biological information, and their regulatory relationships, regulatory effects on fish processes and molecular mechanisms have yet to be verified. In addition, the regulatory role of lncRNAs mediated by miRNAs may also be involved in other biological processes, but the composition and molecular mechanism of the lncRNA-miRNA-mRNA axis need to be confirmed. For example, the differential expression of miRNAs and lncRNAs was also found in tiger pufferfish (*Takifugu rubripes*, *T. rubripes*) of different sexes (109). Compared with female gonads, 79 lncRNAs were upregulated and 51 were downregulated in male gonads, while 3 mature miRNAs were upregulated and 3 mature miRNAs were downregulated. Moreover, several lncRNAs and miRNAs were also predicted to regulate the expression of sex-related genes in *T. rubripes* gonads, such as lnc_000338, lnc_000690, lnc_000370, fru-miR-15b, novel-167, and novel-318 (109). In the central nervous system (CNS) of the weakly electric brown ghost knifefish (*Apteronotus leptorhynchus*, *A. leptorhynchus*), a broad variety of ncRNAs were identified, including lncRNAs, miRNAs, snRNAs, snoRNAs, and other ncRNA sequences (110). These ncRNAs appear to be involved in neurodevelopmental processes such as neurogenesis, neuroregeneration, neuronal differentiation and the neural basis of behavior (110, 112). In addition, in adult zebrafish fed a high-cholesterol diet, the ncRNA regulatory network was shown to be associated with nonalcoholic fatty liver disease (NAFLD) (113). Thirty-two hub lncRNAs, 5 hub miRNAs, and 8 hub mRNAs were identified to be associated with NAFLD-related regulation (113). These results suggest that the regulation of ncRNAs in fish sex determination and differentiation, CNS development, and liver metabolism in fish may be mediated by lncRNA-miRNA-mRNA ceRNA networks, which need to be studied further.

Overall, the available data on the lncRNA-miRNA-mRNA regulatory network in teleost fish, acting as a regulator in physiology and pathology, support its critical role in maintaining cellular homeostasis and functions. However, many regulatory axes are still limited to biological predictions, and more *in vivo* and *in vitro* studies are needed to confirm their composition and function. Additionally, there are still many obstacles that need to be overcome in the research process of noncoding regulatory genes in fish, such as novel ncRNA-based biotechnology tools, screening of core molecular markers, precise intervention strategies, and development of specific ncRNA-related modulators. Therefore, solving the above problems will be an important direction for future fish species research and will further promote the development of selective breeding and sustainable aquaculture.

## Author contributions

ZZ and PL designed the review and contributed to manuscript preparation. ZZ wrote the manuscript. LL, YL and ZG provided technical and administrative support. ZZ and ZW prepared the figures. ZZ, CL, YW and SW revised the manuscript. All authors contributed to the article and approved the submitted version.

## References

[1] Santosh B Varshney A Yadava PK. Non-coding RNAs: Biological functions and applications. Cell Biochem Funct (2015) 33:14–22. doi: 10.1002/cbf.3079 25475931

[2] Djebali S Davis CA Merkel A Dobin A Lassmann T Mortazavi A. Landscape of transcription in human cells. Nature (2012) 489:101–8. doi: 10.1038/nature11233 PMC3684276 22955620

[3] Esteller M. Non-coding RNAs in human disease. Nat Rev Genet (2011) 12:861–74. doi: 10.1038/nrg3074 22094949

[4] Ali SA Peffers MJ Ormseth MJ Jurisica I Kapoor M. The non-coding RNA interactome in joint health and disease. Nat Rev Rheumatol (2021) 17:692–705. doi: 10.1038/s41584-021-00687-y 34588660

[5] Wang ZY Z Xu H Zhang Y Zhang Y. Exosomal noncoding RNAs in central nervous system diseases: Biological functions and potential clinical applications. Front Mol Neurosci (2022) 15:1004221. doi: 10.3389/fnmol.2022.1004221 36438184 PMC9681831

[6] Zuo Y Zhang R Tian J Lv X Li R Li S. Ferroptosis in cancer progression: Role of noncoding RNAs. Int J Biol Sci (2022) 18:1829–43. doi: 10.7150/ijbs.66917 PMC8935228 35342359

[7] Andreassen B. Høyheim, miRNAs associated with immune response in teleost fish. Dev Comp Immunol (2017) 75:77–85. doi: 10.1016/j.dci.2017.02.023 28254620

[8] Bartel DP. MicroRNAs: Genomics, biogenesis, mechanism, and function. Cell (2004) 116:281–97. doi: 10.1016/S0092-8674(04)00045-5 14744438

[9] Lee I Ajay SS Yook JI Kim HS Hong SH Kim NH. New class of microRNA targets containing simultaneous 5'-UTR and 3'-UTR interaction sites. Genome Res (2009) 19:1175–83. doi: 10.1101/gr.089367.108 PMC2704433 19336450

[10] Schnall-Levin M Rissland OS Johnston WK Perrimon N Bartel DP Berger B. Unusually effective microRNA targeting within repeat-rich coding regions of mammalian mRNAs. Genome Res (2011) 21:1395–403. doi: 10.1101/gr.121210.111 PMC3166825 21685129

[11] Friedman RC Farh KKH Burge CB Bartel DP. Most mammalian mRNAs are conserved targets of microRNAs. Genome Res (2009) 19:92–105. doi: 10.1101/gr.082701.108 18955434 PMC2612969

[12] Krol J Loedige I Filipowicz W. The widespread regulation of microRNA biogenesis, function and decay. Nat Rev Genet (2010) 11:597–610. doi: 10.1038/nrg2843 20661255

[13] Loh YH Yi SV Streelman JT. Evolution of microRNAs and the diversification of species. Genome Biol Evol (2011) 3:55–65. doi: 10.1093/gbe/evq085 21169229 PMC3017390

[14] Lim LP Glasner ME Yekta S Burge CB Bartel DP. Vertebrate microRNA genes. Science (2003) 299:1540. doi: 10.1126/science.1080372 12624257

[15] Zhou W Xie Y Li Y Xie M Zhang Z Yang Y. Research progress on the regulation of nutrition and immunity by microRNAs in fish. Fish Shellfish Immunol (2021) 113:1–8. doi: 10.1016/j.fsi.2021.03.011 33766547

[16] Bizuayehu TT Babiak I. MicroRNA in teleost fish. Genome Biol Evol (2014) 6:1911–37. doi: 10.1093/gbe/evu151 PMC4159003 25053657

[17] Best C Ikert H Kostyniuk DJ Craig PM Navarro-Martin L Marandel L. Epigenetics in teleost fish: From molecular mechanisms to physiological phenotypes. Comp Biochem Physiol B Biochem Mol Biol (2018) 224:210–44. doi: 10.1016/j.cbpb.2018.01.006 29369794

[18] Zhou Z Lin Z Pang X Shan P Wang J. MicroRNA regulation of toll-like receptor signaling pathways in teleost fish. Fish Shellfish Immunol (2018) 75:32–40. doi: 10.1016/j.fsi.2018.01.036 29408644

[19] Abo-Al-Ela HG. The emerging regulatory roles of noncoding RNAs in immune function of fish: MicroRNAs versus long noncoding RNAs. Mol Genet Genomics (2021) 296:765–81. doi: 10.1007/s00438-021-01786-x 33904988

[20] Andreassen R Hoyheim B. miRNAs associated with immune response in teleost fish. Dev Comp Immunol (2017) 75:77–85. doi: 10.1016/j.dci.2017.02.023 28254620

[21] Alvi SM Zayed Y Malik R Peng C. The emerging role of microRNAs in fish ovary: A mini review. Gen Comp Endocrinol (2021) 311:113850. doi: 10.1016/j.ygcen.2021.113850 34245767

[22] Xie D Chen C Dong Y You C Wang S Monroig O. Regulation of long-chain polyunsaturated fatty acid biosynthesis in teleost fish. Prog Lipid Res (2021) 82:101095. doi: 10.1016/j.plipres.2021.101095 33741387

[23] Clark MB Mattick JS. Long noncoding RNAs in cell biology. Semin Cell Dev Biol (2011) 22:366–76. doi: 10.1016/j.semcdb.2011.01.001 21256239

[24] Mattick JS. Long noncoding RNAs in cell and developmental biology. Semin Cell Dev Biol (2011) 22:327. doi: 10.1016/j.semcdb.2011.05.002 21621631

[25] Li T Mo X Fu L Xiao B Guo J. Molecular mechanisms of long noncoding RNAs on gastric cancer. Oncotarget (2016) 7:8601–12. doi: 10.18632/oncotarget.6926 PMC4890990 26788991

[26] Zhou Z Wang Z Gao J Lin Z Wang Y Shan P. Noncoding RNA-mediated macrophage and cancer cell crosstalk in hepatocellular carcinoma. Mol Ther Oncolytics (2022) 25:98–120. doi: 10.1016/j.omto.2022.03.002 35506150 PMC9024380

[27] Zhou Z Lin Z He Y Pang X Wang Y Ponnusamy M. The long noncoding RNA D63785 regulates chemotherapy sensitivity in human gastric cancer by targeting miR-422a. Mol Ther Nucleic Acids (2018) 12:405–19. doi: 10.1016/j.omtn.2018.05.024 PMC6036868 30195778

[28] Lin ZJ Zhou ZX Guo H He YQ Pang X Zhang XM. Long noncoding RNA gastric cancer-related lncRNA1 mediates gastric malignancy through miRNA-885-3p and cyclin-dependent kinase 4. Cell Death Dis (2018) 9:607. doi: 10.1038/s41419-018-0643-5 29789536 PMC5964145

[29] Wang KC Chang HY. Molecular mechanisms of long noncoding RNAs. Mol Cell (2011) 43:904–14. doi: 10.1016/j.molcel.2011.08.018 PMC3199020 21925379

[30] Zhang F Zhang L Zhang C. Long noncoding RNAs and tumorigenesis: Genetic associations, molecular mechanisms, and therapeutic strategies. Tumour Biol (2016) 37:163–75. doi: 10.1007/s13277-015-4445-4 26586396

[31] Shetty A Venkatesh T Kabbekodu SP Tsutsumi R Suresh PS. LncRNA-miRNA-mRNA regulatory axes in endometrial cancer: A comprehensive overview. Arch Gynecol Obstet (2022) 306:1431–1447. doi: 10.1007/s00404-022-06423-5 35182183

[32] Barbosa DA Araujo BC Branco GS Simeone AS Hilsdorf AWS Jabes DL. Transcriptomic profiling and microsatellite identification in cobia (Rachycentron canadum), using high-throughput RNA sequencing. Mar Biotechnol (NY) (2022) 24:255–62. doi: 10.1007/s10126-021-10081-0 34855031

[33] Xu H Cao L Sun B Wei Y Liang M. Transcriptomic analysis of potential "lncRNA-mRNA" interactions in liver of the marine teleost cynoglossus semilaevis fed diets with different DHA/EPA ratios. Front Physiol (2019) 10:331. doi: 10.3389/fphys.2019.00331 31001132 PMC6454198

[34] Wang J Fu L Koganti PP Wang L Hand JM Ma H. Identification and functional prediction of Large intergenic noncoding RNAs (lincRNAs) in rainbow trout (Oncorhynchus mykiss). Mar Biotechnol (NY) (2016) 18:271–82. doi: 10.1007/s10126-016-9689-5 26864089

[35] He Z Ye L Yang D Ma Z Deng F He Z. Identification, characterization and functional analysis of gonadal long noncoding RNAs in a protogynous hermaphroditic teleost fish, the ricefield eel (Monopterus albus). BMC Genomics (2022) 23:450. doi: 10.1186/s12864-022-08679-2 35725373 PMC9208217

[36] Song F Wang L Zhu W Dong Z. Long noncoding RNA and mRNA expression profiles following igf3 knockdown in common carp, cyprinus carpio. Sci Data (2019) 6:190024. doi: 10.1038/sdata.2019.24 30778253 PMC6380219

[37] Castro-Arnau J Chauvigne F Gomez-Garrido J Esteve-Codina A Dabad M Alioto T. Developmental RNA-seq transcriptomics of haploid germ cells and spermatozoa uncovers novel pathways associated with teleost spermiogenesis. Sci Rep (2022) 12:14162. doi: 10.1038/s41598-022-18422-2 35986060 PMC9391476

[38] Basu S Hadzhiev Y Petrosino G Nepal C Gehrig J Armant O. The tetraodon nigroviridis reference transcriptome: Developmental transition, length retention and microsynteny of long non-coding RNAs in a compact vertebrate genome. Sci Rep (2016) 6:33210. doi: 10.1038/srep33210 27628538 PMC5024134

[39] Dettleff P Hormazabal E Aedo J Fuentes M Meneses C Molina A. Identification and evaluation of long noncoding RNAs in response to handling stress in red cusk-eel (Genypterus chilensis) *via* RNA-seq. Mar Biotechnol (NY) (2020) 22:94–108. doi: 10.1007/s10126-019-09934-6 31748906

[40] Wang M Jiang S Wu W Yu F Chang WG Li PF. Non-coding RNAs function as immune regulators in teleost fish. Front Immunol (2018) 9:2801. doi: 10.3389/fimmu.2018.02801 30546368 PMC6279911

[41] Sun Q Wang J Wang G Wang H Liu H. Integrated analysis of lncRNA and mRNA in liver of megalobrama amblycephala post aeromonas hydrophila infection. BMC Genomics (2021) 22:653. doi: 10.1186/s12864-021-07969-5 34511071 PMC8435129

[42] Yang N Wang B Yu Z Liu X Fu Q Cao M. Characterization of a novel lncRNA (SETD3-OT) in turbot (Scophthalmus maximus l.). Fish Shellfish Immunol (2020) 102:145–51. doi: 10.1016/j.fsi.2020.04.010 32278113

[43] Liu S Yu T Zhang Y Pan C Cai L Yang M. Integrated analysis of mRNA and long non-coding RNA expression profiles reveals the potential roles of lncRNA-mRNA network in carp macrophage immune regulation. In Vitro Cell Dev Biol Anim (2021) 57:835–47. doi: 10.1007/s11626-021-00610-5 34554377

[44] Valenzuela-Munoz V Pereiro P Alvarez-Rodriguez M Gallardo-Escarate C Figueras A Novoa B. Comparative modulation of lncRNAs in wild-type and rag1-heterozygous mutant zebrafish exposed to immune challenge with spring viraemia of carp virus (SVCV). Sci Rep (2019) 9:14174. doi: 10.1038/s41598-019-50766-0 31578442 PMC6775065

[45] Pereiro P Lama R Moreira R Valenzuela-Munoz V Gallardo-Escarate C Novoa B. Potential involvement of lncRNAs in the modulation of the transcriptome response to nodavirus challenge in European Sea bass (Dicentrarchus labrax l.). Biol (Basel) (2020) 9:165. doi: 10.3390/biology9070165 PMC7407339 32679770

[46] Lee KT Nam JW. Post-transcriptional and translational regulation of mRNA-like long non-coding RNAs by microRNAs in early developmental stages of zebrafish embryos. BMB Rep (2017) 50:226–31. doi: 10.5483/BMBRep.2017.50.4.025 PMC5437968 28320503

[47] Xiu Y Li Y Liu X Li C. Full-length transcriptome sequencing from multiple immune-related tissues of paralichthys olivaceus. Fish Shellfish Immunol (2020) 106:930–7. doi: 10.1016/j.fsi.2020.09.013 32927055

[48] Kern C Wang Y Chitwood J Korf I Delany M Cheng H. Genome-wide identification of tissue-specific long non-coding RNA in three farm animal species. BMC Genomics (2018) 19:684. doi: 10.1186/s12864-018-5037-7 30227846 PMC6145346

[49] Garcia-Perez I Molsosa-Solanas A Perello-Amoros M Sarropoulou E Blasco J Gutierrez J. The emerging role of long non-coding RNAs in development and function of gilthead Sea bream (Sparus aurata) fast skeletal muscle. Cells (2022) 11:428. doi: 10.3390/cells11030428 35159240 PMC8834446

[50] Chen Y Wan S Li Q Dong X Diao J Liao Q. Genome-wide integrated analysis revealed functions of lncRNA-miRNA-mRNA interaction in growth of intermuscular bones in megalobrama amblycephala. Front Cell Dev Biol (2020) 8:603815. doi: 10.3389/fcell.2020.603815 33614620 PMC7891300

[51] Papadaki M Kaitetzidou E Mylonas CC Sarropoulou E. Non-coding RNA expression patterns of two different teleost gonad maturation stages. Mar Biotechnol (NY) (2020) 22:683–95. doi: 10.1007/s10126-020-09991-2 32876760

[52] Cheng J Yang F Liu S Zhao H Lu W Zhang Q. Transcriptomic analysis reveals functional interaction of mRNA-lncRNA-miRNA in steroidogenesis and spermatogenesis of gynogenetic Japanese flounder (Paralichthys olivaceus). Biol (Basel) (2022) 11:213. doi: 10.3390/biology11020213 PMC8869744 35205081

[53] Baumann L Holbech H Keiter S Kinnberg KL Knorr S Nagel T. The maturity index as a tool to facilitate the interpretation of changes in vitellogenin production and sex ratio in the fish sexual development test. Aquat Toxicol (2013) 128:34–42. doi: 10.1016/j.aquatox.2012.11.016 23261669

[54] Tang Y Xin G Zhao L Huang L Qin Y Su Y. Novel insights into host-pathogen interactions of large yellow croakers (Larimichthys crocea) and pathogenic bacterium pseudomonas plecoglossicida using time-resolved dual RNA-seq of infected spleens. Zoological Res (2020) 41:314–327. doi: 10.24272/j.issn.2095-8137.2020.035. PMC7231473 32242645

[55] Yuan B Zhao L Zhuang Z Wang X Fu Q Huang H. Transcriptomic and metabolomic insights into the role of the flgK gene in the pathogenicity of pseudomonas plecoglossicida to orange-spotted grouper (Epinephelus coioides). Zoological Res (2022) 43:952–65. doi: 10.24272/j.issn.2095-8137.2022.216 PMC9700492 36208122

[56] Watanabe M Abe N Oshikiri Y Stanbridge EJ Kitagawa T. Selective growth inhibition by glycogen synthase kinase-3 inhibitors in tumorigenic HeLa hybrid cells is mediated through NF-kappaB-dependent GLUT3 expression. Oncogenesis (2012) 1:e21. doi: 10.1038/oncsis.2012.21 23552737 PMC3412655

[57] Zhang Y Cui C Lai ZC. The defender against apoptotic cell death 1 gene is required for tissue growth and efficient n-glycosylation in drosophila melanogaster. Dev Biol (2016) 420:186–95. doi: 10.1016/j.ydbio.2016.09.021 27693235

[58] Zheng W Chang R Luo Q Liu G Xu T. The long noncoding RNA MIR122HG is a precursor for miR-122-5p and negatively regulates the TAK1-induced innate immune response in teleost fish. J Biol Chem (2022) 298:101773. doi: 10.1016/j.jbc.2022.101773 35218771 PMC8935508

[59] Sarangdhar MA Chaubey D Srikakulam N Pillai B. Parentally inherited long non-coding RNA cyrano is involved in zebrafish neurodevelopment. Nucleic Acids Res (2018) 46:9726–35. doi: 10.1093/nar/gky628 PMC6182166 30011017

[60] Gomez-Nicola D Pallas-Bazarra N Valle-Argos B Nieto-Sampedro M. CCR7 is expressed in astrocytes and upregulated after an inflammatory injury. J Neuroimmunol (2010) 227:87–92. doi: 10.1016/j.jneuroim.2010.06.018 20638137

[61] Wang P Zhao Z Zhang Z Cai Z Liao J Tan Q. Genome-wide identification and analysis of NPR family genes in brassica juncea var. tumida. Gene (2021) 769:145210. doi: 10.1016/j.gene.2020.145210 33069807

[62] Wu S Zhang J Liu B Huang Y Li S Wen H. Identification and characterization of lncRNAs related to the muscle growth and development of Japanese flounder (Paralichthys olivaceus). Front Genet (2020) 11:1034. doi: 10.3389/fgene.2020.01034 33033494 PMC7510837

[63] Tang R Luo G Zhao L Huang L Qin Y Xu X. The effect of a LysR-type transcriptional regulator gene of pseudomonas plecoglossicida on the immune responses of epinephelus coioides. Fish Shellfish Immunol (2019) 89:420–7. doi: 10.1016/j.fsi.2019.03.051 30974221

[64] Luo G Zhao L Xu X Qin Y Huang L Su Y. Integrated dual RNA-seq and dual iTRAQ of infected tissue reveals the functions of a diguanylate cyclase gene of pseudomonas plecoglossicida in host-pathogen interactions with epinephelus coioides. Fish Shellfish Immunol (2019) 95:481–90. doi: 10.1016/j.fsi.2019.11.008 31698069

[65] Shen Y Liang W Lin Y Yang H Chen X Feng P. Single molecule real-time sequencing and RNA-seq unravel the role of long non-coding and circular RNA in the regulatory network during Nile tilapia (Oreochromis niloticus) infection with streptococcus agalactiae. Fish Shellfish Immunol (2020) 104:640–53. doi: 10.1016/j.fsi.2020.06.015 32544555

[66] Li L Jia X Liu Y He Y Pang Y Shen Y. lncRNA-SUMO3 and lncRNA-HDMO13 modulate the inflammatory response by binding miR-21 and miR-142a-3p in grass carp. Dev Comp Immunol (2021) 121:104082. doi: 10.1016/j.dci.2021.104082 33785433

[67] Ramos E Cardoso AL Brown J Marques DF Fantinatti BE Cabral-de-Mello DC. The repetitive DNA element BncDNA, enriched in the b chromosome of the cichlid fish astatotilapia latifasciata, transcribes a potentially noncoding RNA. Chromosoma (2017) 126:313–23. doi: 10.1007/s00412-016-0601-x 27169573

[68] He Y Huang CX Chen N Wu M Huang Y Liu H. The zebrafish miR-125c is induced under hypoxic stress *via* hypoxia-inducible factor 1 alpha and functions in cellular adaptations and embryogenesis. Oncotarget (2017) 8:73846–59. doi: 10.18632/oncotarget.17994 PMC5650306 29088751

[69] Zhang Z Lau SW Zhang L Ge W. Disruption of zebrafish follicle-stimulating hormone receptor (fshr) but not luteinizing hormone receptor (lhcgr) gene by TALEN leads to failed follicle activation in females followed by sexual reversal to males. Endocrinology (2015) 156:3747–62. doi: 10.1210/en.2015-1039 25993524

[70] Fan K Shen Y Xu X Tao L Bao T Li J. LncRNA-WAS and lncRNA-C8807 interact with miR-142a-3p to regulate the inflammatory response in grass carp. Fish Shellfish Immunol (2021) 111:201–7. doi: 10.1016/j.fsi.2021.02.003 33582280

[71] Wu J Zhang L Feng Y Khadka B Fang Z Liu J. HDAC8 promotes daunorubicin resistance of human acute myeloid leukemia cells *via* regulation of IL-6 and IL-8. Biol Chem (2021) 402:461–8. doi: 10.1515/hsz-2020-0196 33938176

[72] Xia YQ Cheng JX Liu YF Li CH Liu Y Liu PF. Genome-wide integrated analysis reveals functions of lncRNA-miRNA-mRNA interactions in Atlantic salmon challenged by aeromonas salmonicida. Genomics (2022) 114:328–39. doi: 10.1016/j.ygeno.2021.12.013 34933071

[73] Gao C Cai X Ma L Li C. Identification of mRNA-miRNA-lncRNA regulatory network associated with the immune response to aeromonas salmonicides infection in the black rockfish (Sebastes schlegelii). Dev Comp Immunol (2022) 130:104357. doi: 10.1016/j.dci.2022.104357 35090885

[74] Chu Q Xu T Zheng W Chang R Zhang L. Long noncoding RNA MARL regulates antiviral responses through suppression miR-122-dependent MAVS downregulation in lower vertebrates. PloS Pathog (2020) 16:e1008670. doi: 10.1371/journal.ppat.1008670 32678830 PMC7390449

[75] Samsing F Wynne JW Valenzuela-Munoz V Valenzuela-Miranda D Gallardo-Escarate C Alexandre PA. Competing endogenous RNA-networks reveal key regulatory microRNAs involved in the response of Atlantic salmon to a novel orthomyxovirus. Dev Comp Immunol (2022) 132:104396. doi: 10.1016/j.dci.2022.104396 35304180

[76] Avery JC Hoffmann PR. Selenium, selenoproteins, and immunity. Nutrients (2018) 10:1203. doi: 10.3390/nu10091203 30200430 PMC6163284

[77] Zhou CQ Ka W Yuan WK Wang JL. The effect of acute heat stress on the innate immune function of rainbow trout based on the transcriptome. J Therm Biol (2021) 96:102834. doi: 10.1016/j.jtherbio.2021.102834 33627272

[78] Wu S Huang J Li Y Liu Z Zhao L. Integrated analysis of lncRNA and circRNA mediated ceRNA regulatory networks in skin reveals innate immunity differences between wild-type and yellow mutant rainbow trout (Oncorhynchus mykiss). Front Immunol (2022) 13:802731. doi: 10.3389/fimmu.2022.802731 35655786 PMC9152293

[79] Arun G Diermeier SD Spector DL. Therapeutic targeting of long non-coding RNAs in cancer. Trends Mol Med (2018) 24:257–77. doi: 10.1016/j.molmed.2018.01.001 PMC5840027 29449148

[80] Ye W Duan Y Zhang W Cheng Y Shi M Xia XQ. Comprehensive analysis of hub mRNA, lncRNA and miRNA, and associated ceRNA networks implicated in grass carp (Ctenopharyngodon idella) growth traits. Genomics (2021) 113:4004–14. doi: 10.1016/j.ygeno.2021.10.001 34614437

[81] Leiva F Rojas-Herrera M Reyes D Bravo S Garcia KK Moya J. Identification and characterization of miRNAs and lncRNAs of coho salmon (Oncorhynchus kisutch) in normal immune organs. Genomics (2020) 112:45–54. doi: 10.1016/j.ygeno.2019.07.015 31376527

[82] Ali A Al-Tobasei R Kenney B Leeds TD Salem M. Integrated analysis of lncRNA and mRNA expression in rainbow trout families showing variation in muscle growth and fillet quality traits. Sci Rep (2018) 8:12111. doi: 10.1038/s41598-018-30655-8 30108261 PMC6092380

[83] Danos N Ward AB. The homology and origins of intermuscular bones in fishes: phylogenetic or biomechanical determinants? Biol J Linn Soc (2012) 106:607–22. doi: 10.1111/j.1095-8312.2012.01893.x

[84] Kocher TD. Adaptive evolution and explosive speciation: The cichlid fish model. Nat Rev Genet (2004) 5:288–98. doi: 10.1038/nrg1316 15131652

[85] Ferre LE Medesani DA Garcia CF Grodzielski M Rodriguez EM. Vitellogenin levels in hemolymph, ovary and hepatopancreas of the freshwater crayfish cherax quadricarinatus (Decapoda: Parastacidae) during the reproductive cycle. Rev Biol Trop (2012) 60:253–61. doi: 10.15517/rbt.v60i1.2759 22458222

[86] Mang Q Hou J Han T Wang G Wang Y Liu Y. The effect of infertility on the liver structure, endocrinology, and gene network in Japanese flounder. Anim (Basel) (2021) 11:936. doi: 10.3390/ani11040936 PMC8066618 33806167

[87] Pang Y Li L Yang Y Shen Y Xu X Li J. LncRNA-ANAPC2 and lncRNA-NEFM positively regulates the inflammatory response *via* the miR-451/npr2/ hdac8 axis in grass carp. Fish Shellfish Immunol (2022) 128:1–6. doi: 10.1016/j.fsi.2022.07.014 35843524

[88] Zhao D Deng SC Ma Y Hao YH Jia ZH. miR-221 alleviates the inflammatory response and cell apoptosis of neuronal cell through targeting TNFAIP2 in spinal cord ischemia-reperfusion. Neuroreport (2018) 29:655–60. doi: 10.1097/WNR.0000000000001013 29596155

[89] Xiu Y Li Y Liu X Su L Zhou S Li C. Identification and characterization of long non-coding RNAs in the intestine of olive flounder (Paralichthys olivaceus) during edwardsiella tarda infection. Front Immunol (2021) 12:623764. doi: 10.3389/fimmu.2021.623764 33868240 PMC8044400

[90] Yan Q Si J Cui X Peng H Jing M Chen X. GmDAD1, a conserved defender against cell death 1 (DAD1) from soybean, positively regulates plant resistance against phytophthora pathogens. Front Plant Sci (2019) 10:107. doi: 10.3389/fpls.2019.00107 30800138 PMC6376896

[91] Zhang B Luo G Zhao L Huang L Qin Y Su Y. Integration of RNAi and RNA-seq uncovers the immune responses of epinephelus coioides to L321_RS19110 gene of pseudomonas plecoglossicida. Fish Shellfish Immunol (2018) 81:121–9. doi: 10.1016/j.fsi.2018.06.051 30006040

[92] Sun Y Luo G Zhao L Huang L Qin Y Su Y. Integration of RNAi and RNA-seq reveals the immune responses of epinephelus coioides to sigX gene of pseudomonas plecoglossicida. Front Immunol (2018) 9:1624. doi: 10.3389/fimmu.2018.01624 30061893 PMC6054955

[93] Ning X Sun L. Identification and characterization of immune-related lncRNAs and lncRNA-miRNA-mRNA networks of paralichthys olivaceus involved in vibrio anguillarum infection. BMC Genomics (2021) 22:447. doi: 10.1186/s12864-021-07780-2 34130627 PMC8204505

[94] Zheng W Chu Q Xu T. Long noncoding RNA IRL regulates NF-kappaB-mediated immune responses through suppression of miR-27c-3p-dependent IRAK4 downregulation in teleost fish. J Biol Chem (2021) 296:100304. doi: 10.1016/j.jbc.2021.100304 33465375 PMC7949060

[95] Chu Q Xu T Zheng W Chang R Zhang L. Long noncoding RNA AANCR modulates innate antiviral responses by blocking miR-210-dependent MITA downregulation in teleost fish, miichthys miiuy. Sci China Life Sci (2021) 64:1131–48. doi: 10.1007/s11427-020-1789-5 32997329

[96] Zheng W Chu Q Xu T. The long noncoding RNA NARL regulates immune responses *via* microRNA-mediated NOD1 downregulation in teleost fish. J Biol Chem (2021) 296:100414. doi: 10.1016/j.jbc.2021.100414 33581111 PMC7966872

[97] Chang R Zheng W Sun Y Geng S Xu T. Long noncoding RNA MIR2187HG suppresses TBK1-mediated antiviral signaling by deriving miR-2187-3p in teleost fish. J Virol (2022) 96:e0148421. doi: 10.1128/JVI.01484-21 34643431 PMC8754209

[98] Li BJ Jiang DL Meng ZN Zhang Y Zhu ZX Lin HR. Genome-wide identification and differentially expression analysis of lncRNAs in tilapia. BMC Genomics (2018) 19:729. doi: 10.1186/s12864-018-5115-x 30286721 PMC6172845

[99] Corsini LR Bronte G Terrasi M Amodeo V Fanale D Fiorentino E. The role of microRNAs in cancer: diagnostic and prognostic biomarkers and targets of therapies. Expert Opin Ther Targets (2012) 16 Suppl 2:S103–109. doi: 10.1517/14728222.2011.650632 22443195

[100] Singh D Khan MA Siddique HR. Emerging role of long non-coding RNAs in cancer chemoresistance: Unravelling the multifaceted role and prospective therapeutic targeting. Mol Biol Rep (2020) 47:5569–85. doi: 10.1007/s11033-020-05609-x 32601922

[101] Shalaby KE Aouida M Gupta V Abdesselem H El-Agnaf OMA. Development of non-viral vectors for neuronal-targeted delivery of CRISPR-Cas9 RNA-proteins as a therapeutic strategy for neurological disorders. Biomater Sci (2022) 10:4959–77. doi: 10.1039/D2BM00368F 35880637

[102] Bhan A Soleimani M Mandal SS. Long noncoding RNA and cancer: A new paradigm. Cancer Res (2017) 77:3965–81. doi: 10.1158/0008-5472.CAN-16-2634 PMC8330958 28701486

[103] Yan Y Liu XY Lu A Wang XY Jiang LX Wang JC. Non-viral vectors for RNA delivery. J Control Release (2022) 342:241–79. doi: 10.1016/j.jconrel.2022.01.008 PMC8743282 35016918

[104] Nunez-Acuna G Detree C Gallardo-Escarate C Goncalves AT. Functional diets modulate lncRNA-coding RNAs and gene interactions in the intestine of rainbow trout oncorhynchus mykiss. Mar Biotechnol (NY) (2017) 19:287–300. doi: 10.1007/s10126-017-9750-z 28500613

[105] Cui Y Wan H Zhang X. miRNA in food simultaneously controls animal viral disease and human tumorigenesis. Mol Ther Nucleic Acids (2021) 23:995–1006. doi: 10.1016/j.omtn.2021.01.011 33614246 PMC7868940

[106] Chen Y Zhang S Cao J Zhang X. Shrimp antiviral mja-miR-35 targets CHI3L1 in human M2 macrophages and suppresses breast cancer metastasis. Front Immunol (2018) 9:2071. doi: 10.3389/fimmu.2018.02071 30258444 PMC6143669

[107] Chen Q Zhang F Dong L Wu H Xu J Li H. SIDT1-dependent absorption in the stomach mediates host uptake of dietary and orally administered microRNAs. Cell Res (2021) 31:247–58. doi: 10.1038/s41422-020-0389-3 PMC8026584 32801357

[108] Yang L Xu Z Zeng H Sun N Wu B Wang C. FishDB: an integrated functional genomics database for fishes. BMC Genomics (2020) 21:801. doi: 10.1186/s12864-020-07159-9 33203359 PMC7670658

[109] Yan HW Liu Q Jiang JM Shen XF Zhang L Yuan Z. Identification of sex differentiation-related microRNA and long non-coding RNA in takifugu rubripes gonads. Sci Rep-Uk (2021) 11:7459. doi: 10.1038/s41598-021-83891-w PMC8018949 33811216

[110] Salisbury JP Sirbulescu RF Moran BM Auclair JR Zupanc GK Agar JN. The central nervous system transcriptome of the weakly electric brown ghost knifefish (Apteronotus leptorhynchus): de novo assembly, annotation, and proteomics validation. BMC Genomics (2015) 16:166. doi: 10.1186/s12864-015-1354-2 25879418 PMC4424500

[111] Morris KV Mattick JS. The rise of regulatory RNA. Nat Rev Genet (2014) 15:423–37. doi: 10.1038/nrg3722 PMC4314111 24776770

[112] Liu W Liu X Wu C Jiang L. Transcriptome analysis demonstrates that long noncoding RNA is involved in the hypoxic response in larimichthys crocea. Fish Physiol Biochem (2018) 44:1333–47. doi: 10.1007/s10695-018-0525-x 29948448

[113] An HJ Lee YJ Choe CP Cho HK Song DH. Long noncoding RNAs associated with nonalcoholic fatty liver disease in a high cholesterol diet adult zebrafish model. Sci Rep (2021) 11:23005. doi: 10.1038/s41598-021-02455-0 34837012 PMC8626429

